# Supplementary figures and images for: Enhanced cytotoxicity of T-DM1 in HER2-low carcinomas via autophagy inhibition
Source: PLoS One. 2025 May 2;20(5):e0322029. doi: 10.1371/journal.pone.0322029 (PMC12047817; doi:10.1371/journal.pone.0322029)

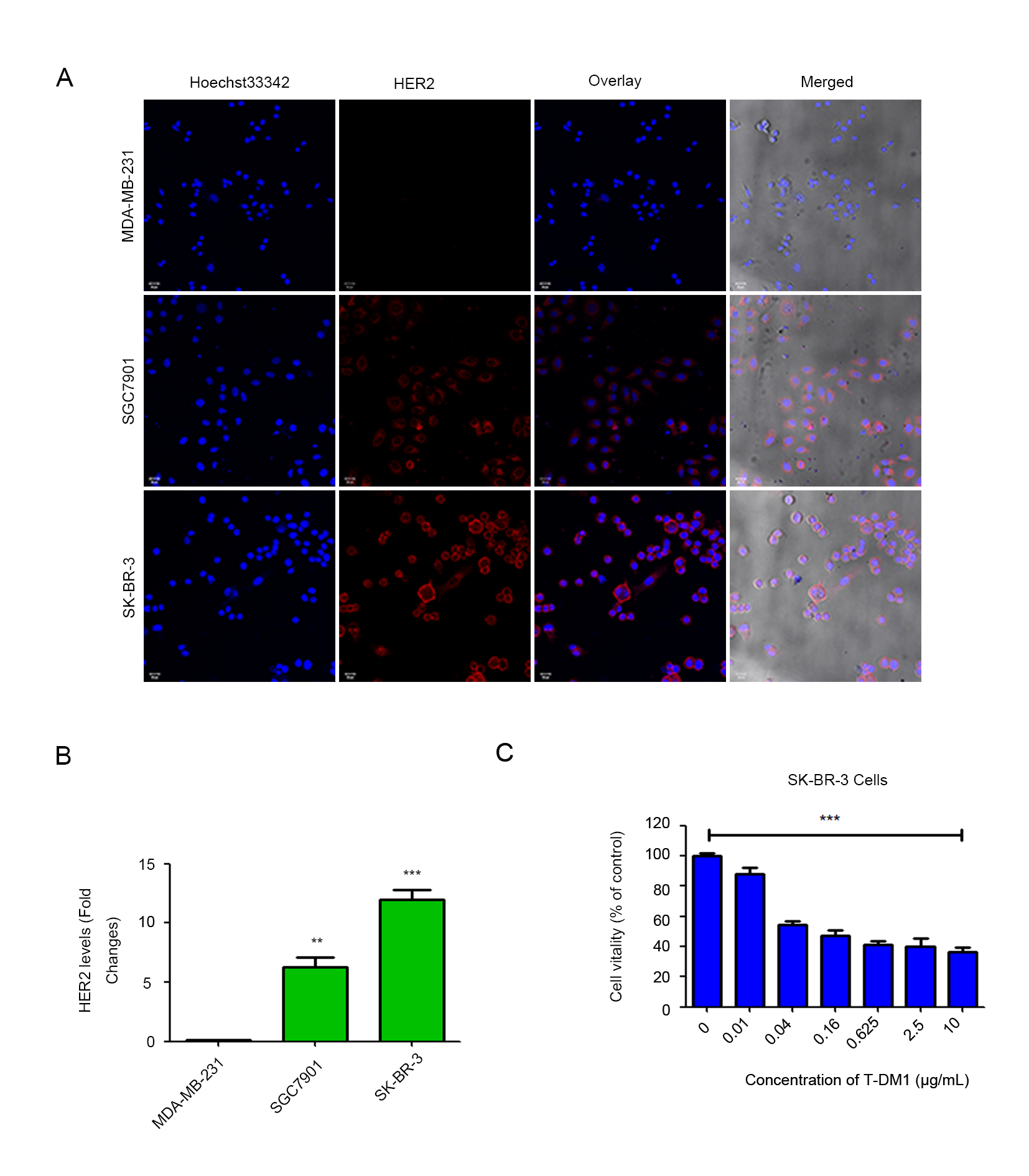

Supplement: Supplementary Figure S1 — (A) The expression of HER2 was examined by immunofluorescence in SGC7901, HER2-negative MDA-MB-231 and HER2 + SK-BR-3 cells using a confocal microscope. (B) The brightness values of HER2 red dye staining were estimated using ImageJ software and the densitometric values were normalized to the corresponding values of the vehicle group. The values of the vehicle group were set as 1.0 (n = 3 independent experiments; mean ± S.D.; **P < 0.01 and ***P < 0.001). (C) CCK-8 assays were used to evaluate the survival rates of SK-BR-3 cells (HER2-high Breast cancer cells) treated with T-DM1 in a concentration-dependent manner for 72 hours (mean ± S.D.; ***P < 0.001, n = 3). (TIF) [file pone.0322029.s001.tif]

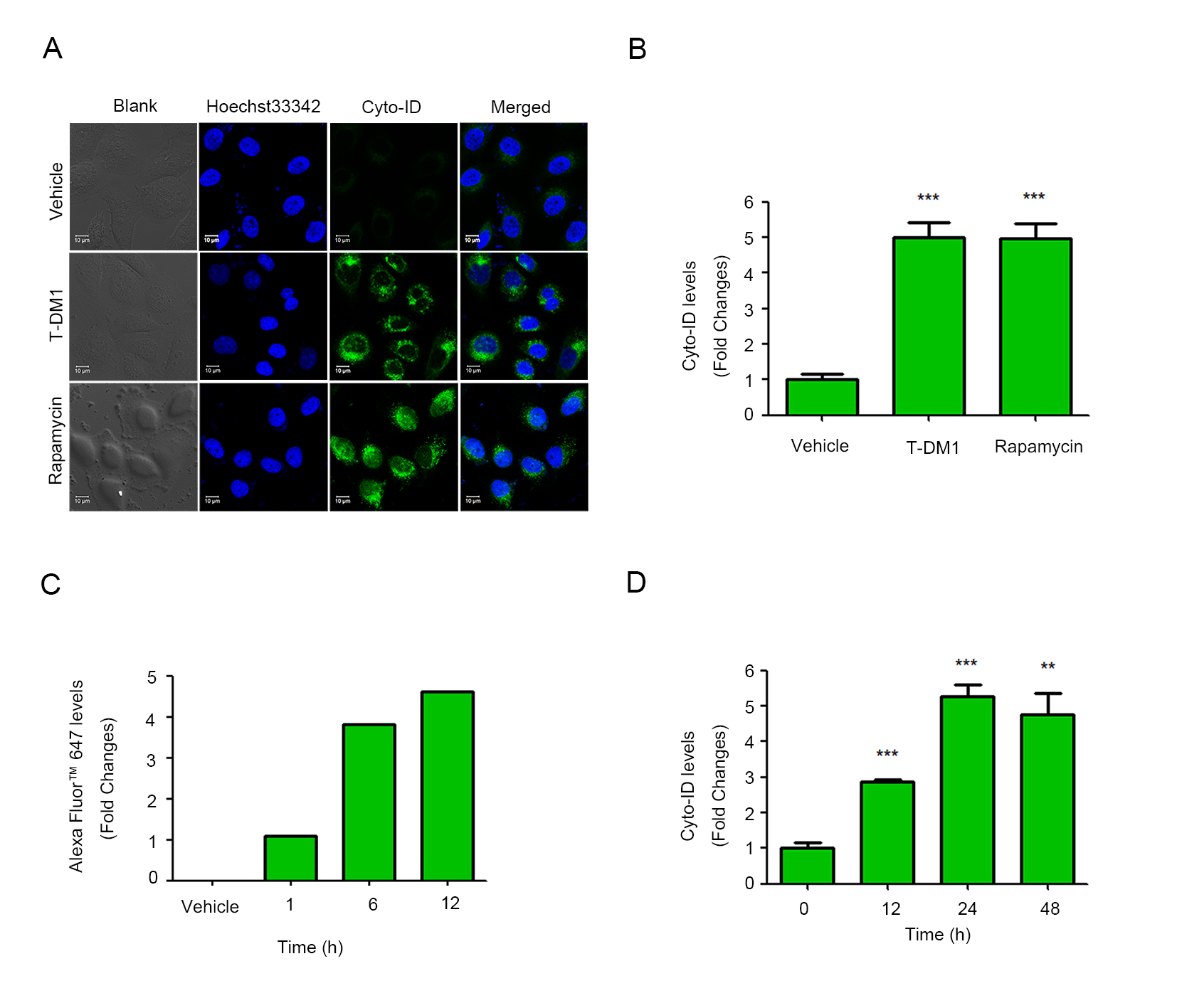

Supplement: Supplementary Figure S2 — (A) Confocal immunofluorescence was used to verify autophagosome staining with Cyto-ID fluorescent dye in SGC7901 cells after treatment with T-DM1 for 24 hours, with the group incubated with rapamycin serving as the control. (B) The brightness values of Cyto-ID green dye staining were estimated using ImageJ software and the densitometric values were normalized to the corresponding values of the vehicle group. The values of the vehicle group were set as 1.0 (n = 3 independent experiments; mean ± S.D.; ***P < 0.001). (C) The brightness values of the Alexa Fluor™ 647 were evaluated using ImageJ software, and the values were normalized to the corresponding values of the vehicle group. (D) The brightness values of autophagic flux were evaluated using ImageJ software, and the values were normalized to the corresponding values of the vehicle group. The values of the vehicle group were set as 1.0 (n = 3 independent experiments; mean ± S.D.; **P < 0.01 and ***P < 0.001). (TIF) [file pone.0322029.s002.tif]

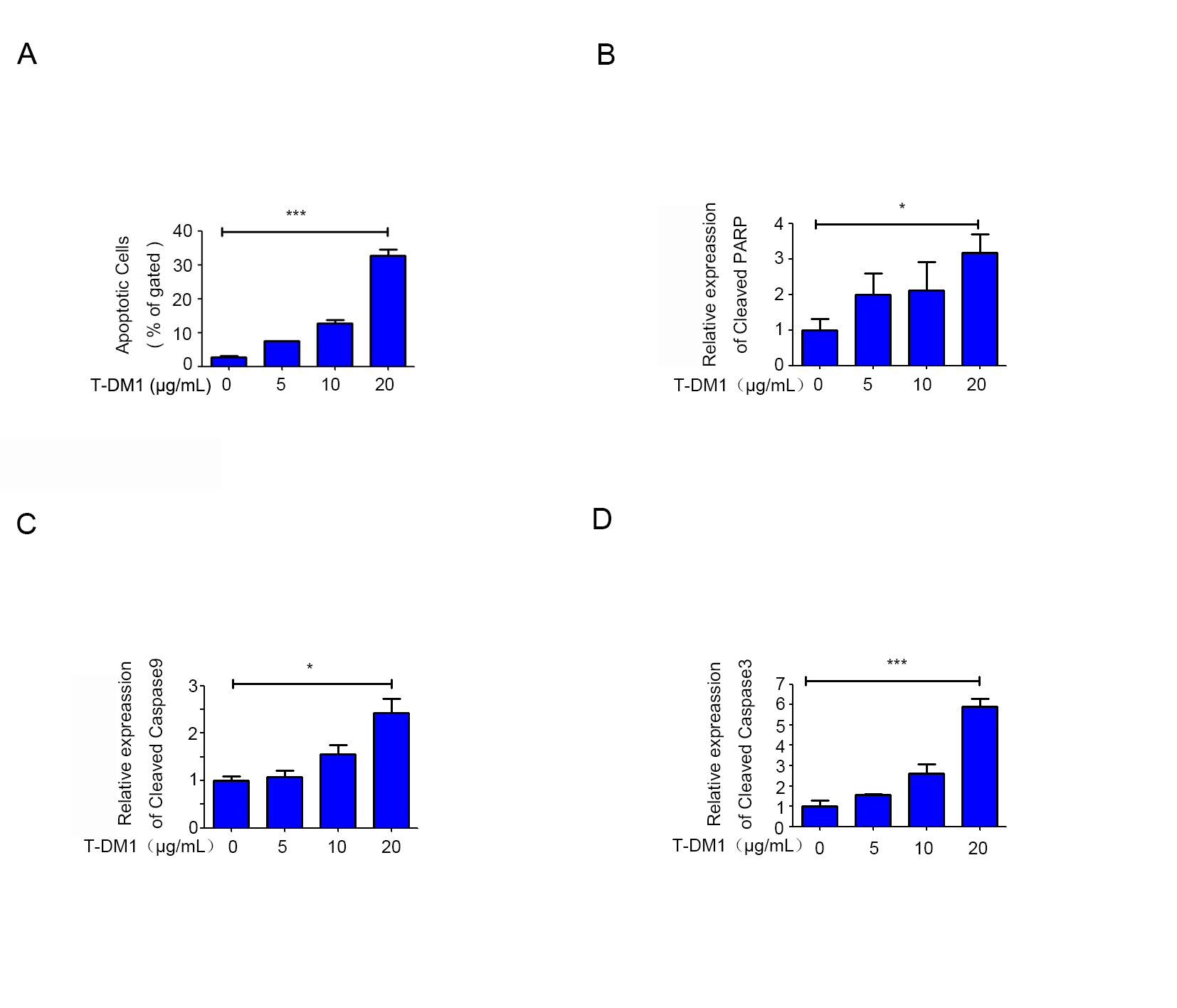

Supplement: Supplementary Figure S3 — (A) Apoptotic cells (the Annexin V + and PI- cells) examined by FCM for the indicated time (48 h) were counted and analyzed, with results shown in bar charts (n = 3; mean ± S.D.; ***P < 0.001). (B-D) Densitometric values of the expression of apoptotic proteins (cleaved PARP, cleaved caspase 9, and cleaved caspase 3) after SGC7901 cells treated with T-DM1 for 48 hours were calculated using ImageJ software and normalized to the value of the corresponding vehicle bands. The values of the vehicle group were adjusted to 1.0 and are shown as the mean ± S.D. Student’s t-test was used to analyze the data. *P < 0.05 and ***P < 0.001, n = 3. (TIF) [file pone.0322029.s003.tif]

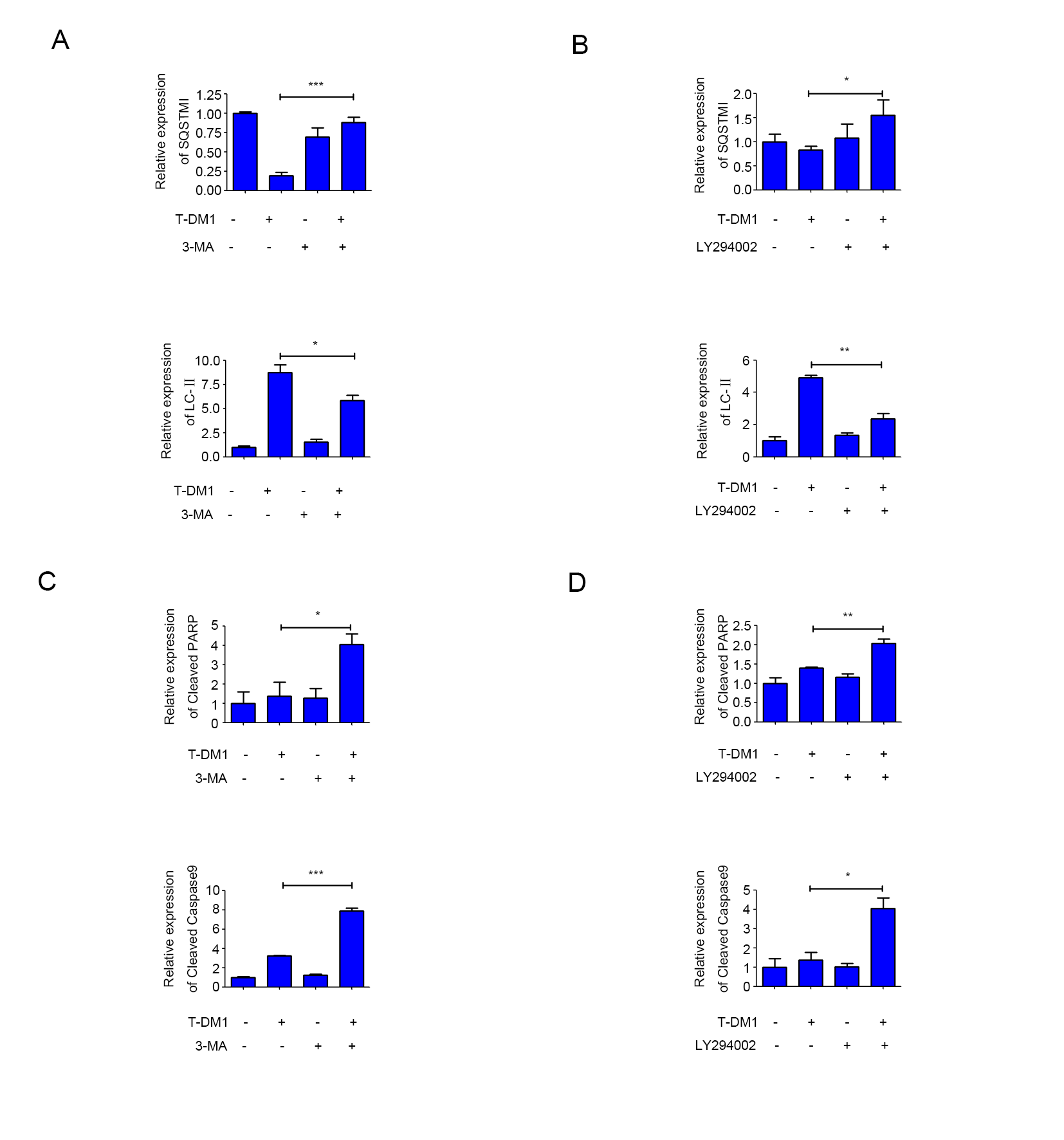

Supplement: Supplementary Figure S4 — (A-D) Densitometric values of SQSTM1, LC3-Ⅱ, cleaved PARP, and cleaved caspase 9 in SGC7901 cells treated with T-DM1 and autophagy inhibitor (3-MA or LY294002) were analyzed using ImageJ software (n = 3; mean ± S.D.; *P < 0.05, **P < 0.01, and ***P < 0.001). (TIF) [file pone.0322029.s004.tif]

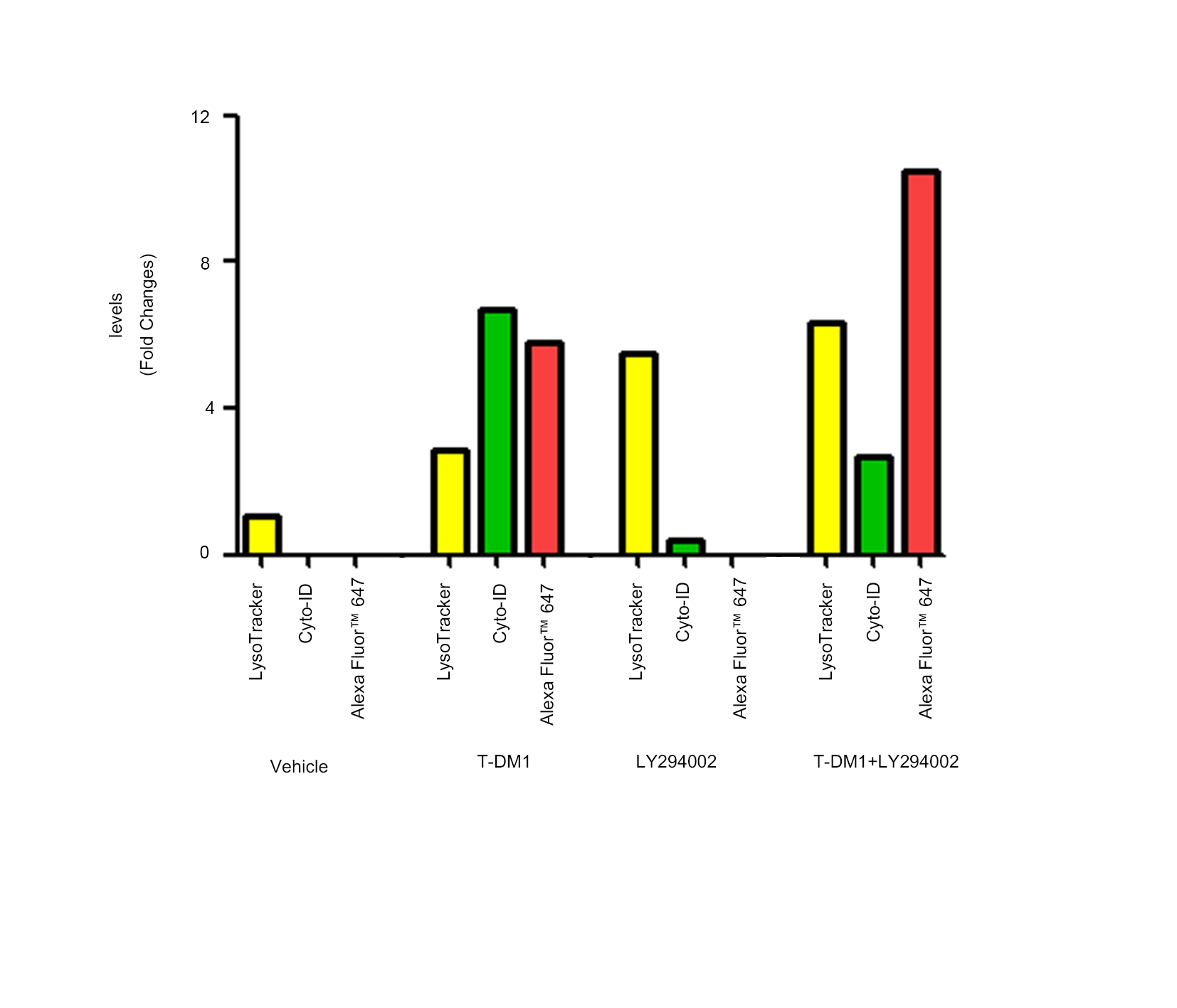

Supplement: Supplementary Figure S5 — The brightness values of the Alexa Fluor™ 647 visualized by red fluorescence, Cyto-ID visualized by fluorescence and LysoTracker exhibited yellow fluorescence were evaluated using ImageJ software, and the values were normalized to the corresponding values of the vehicle group. (TIF) [file pone.0322029.s005.tif]

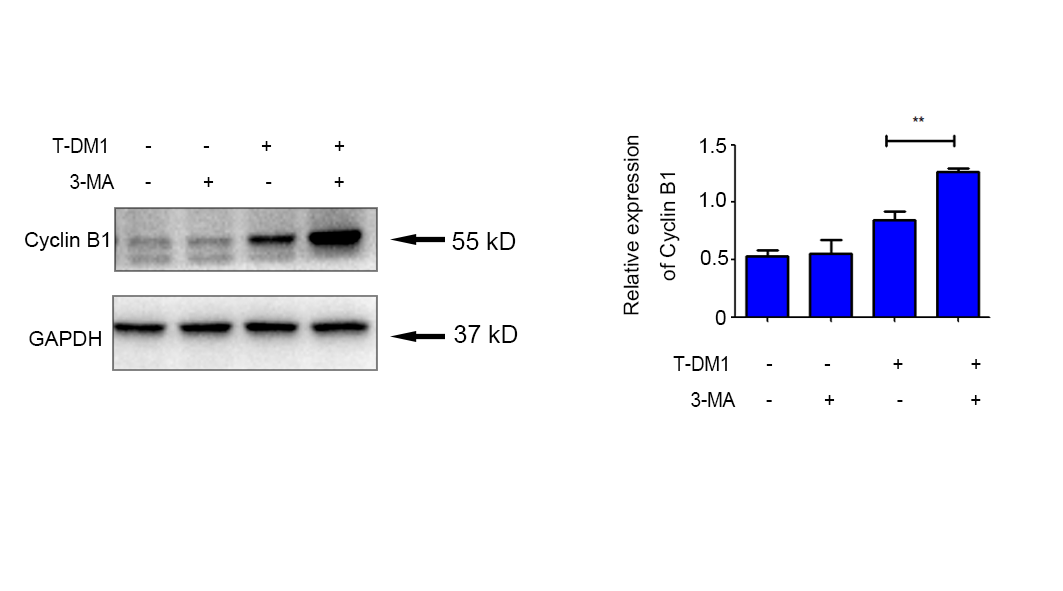

Supplement: Supplementary Figure S6 — SGC7901 cells were incubated with T-DM1 and 3-MA for 48 hours, and cell lysates were analyzed by immunoblotting to determine the expression levels of the CycinB1. The densitometric values of SGC7901 cell samples were evaluated using ImageJ software and normalized to the corresponding values of the vehicle group. The values of the vehicle group were adjusted to 1.0 (mean ± S.D.; *P < 0.05; **P < 0.01 versus vehicle, n = 3). (TIF) [file pone.0322029.s006.tif]

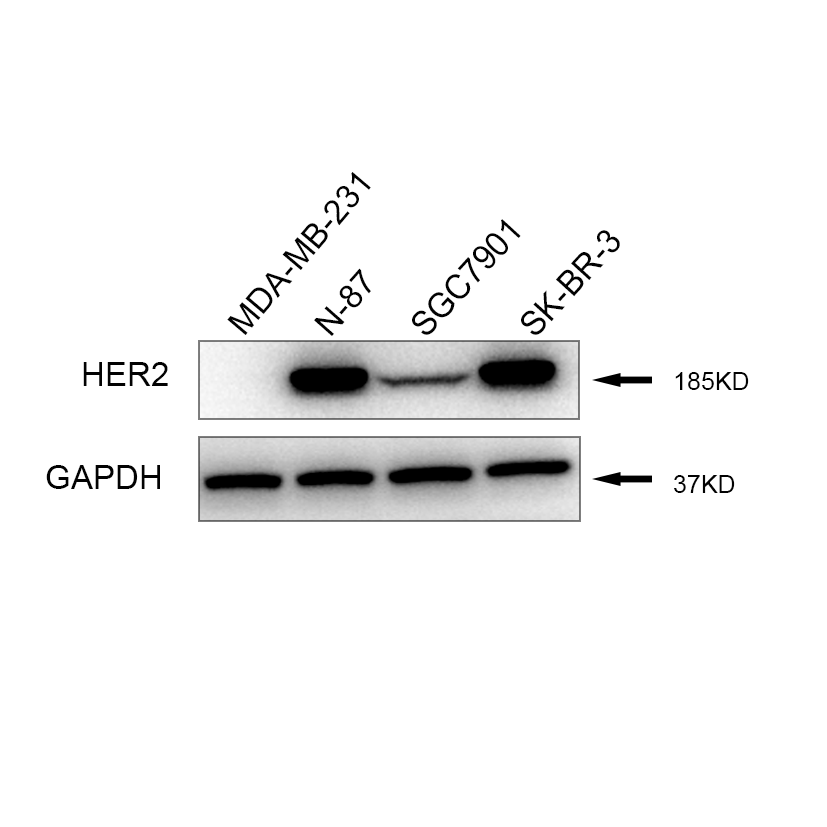

Supplement: Supplementary Figure S7 — The cell lysates of MDA-MB-231 (HER2-negative Breast cancer cell line), N-87 cells (HER2-positive GC cancer cell line), SGC7901 and SK-BR-3 cells (HER2-positive Breast cancer cell line) were analyzed by immunoblotting to determine the expression levels of the HER2. (TIF) [file pone.0322029.s007.tif]

Figure 2

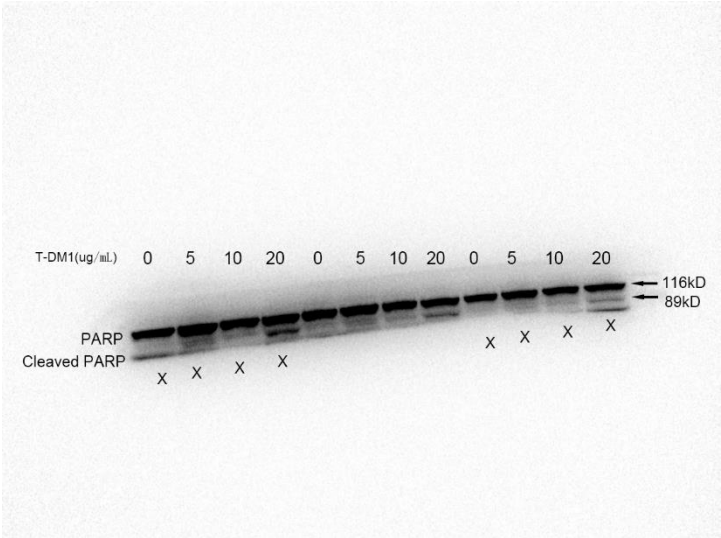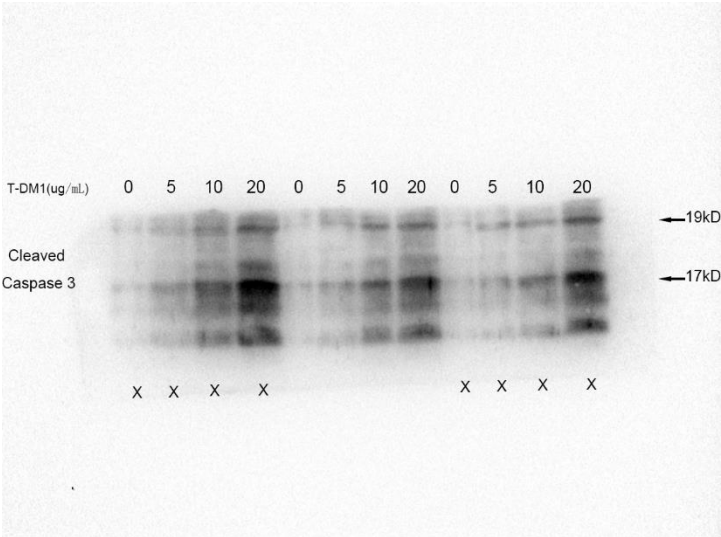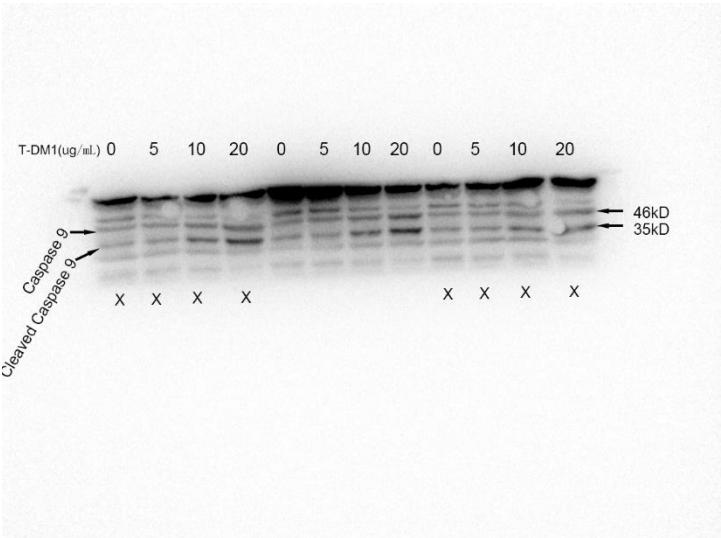

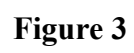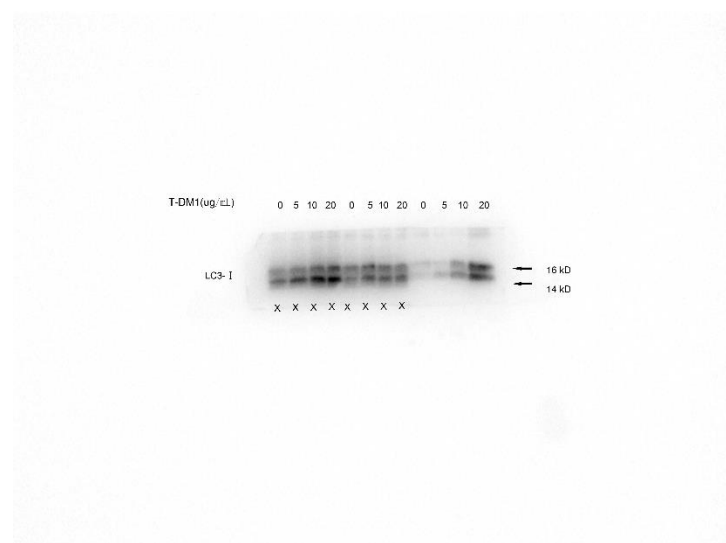



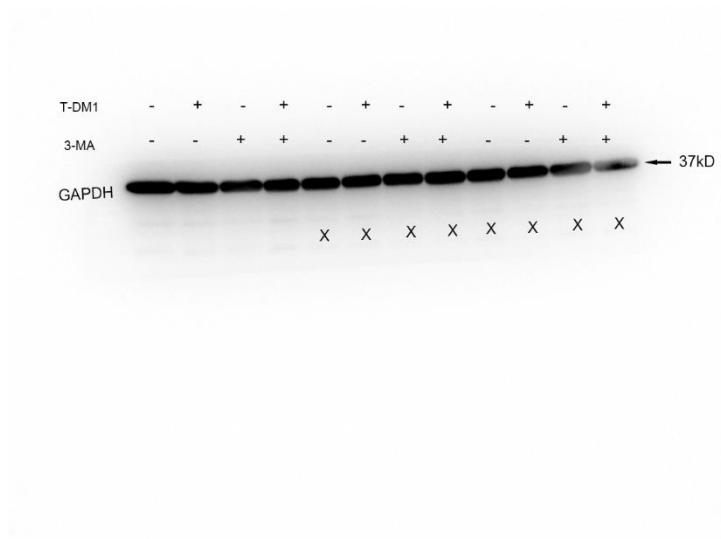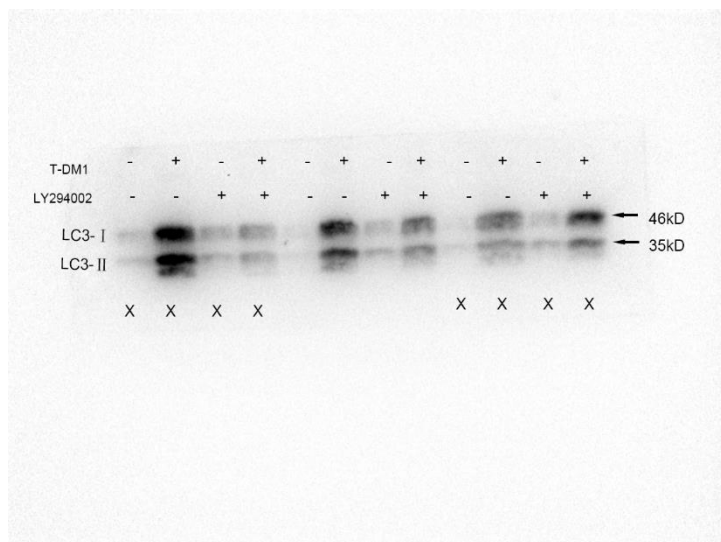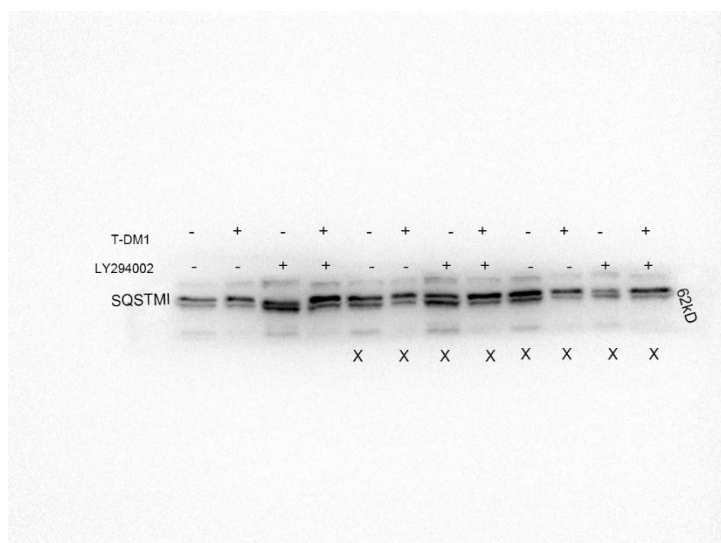

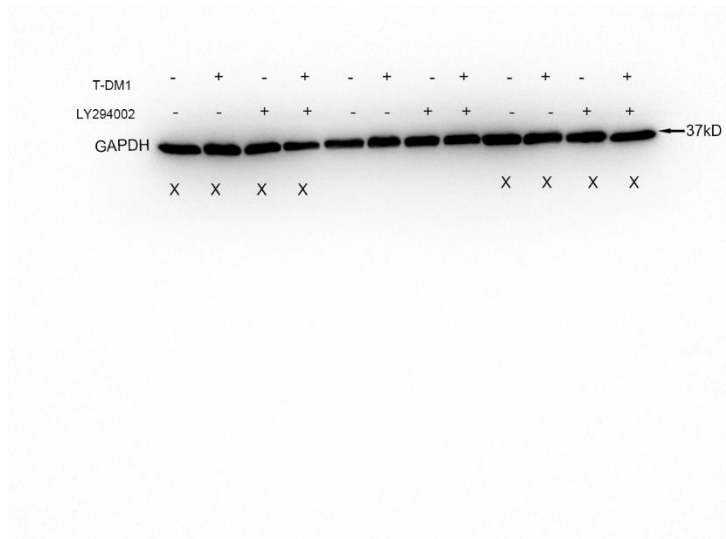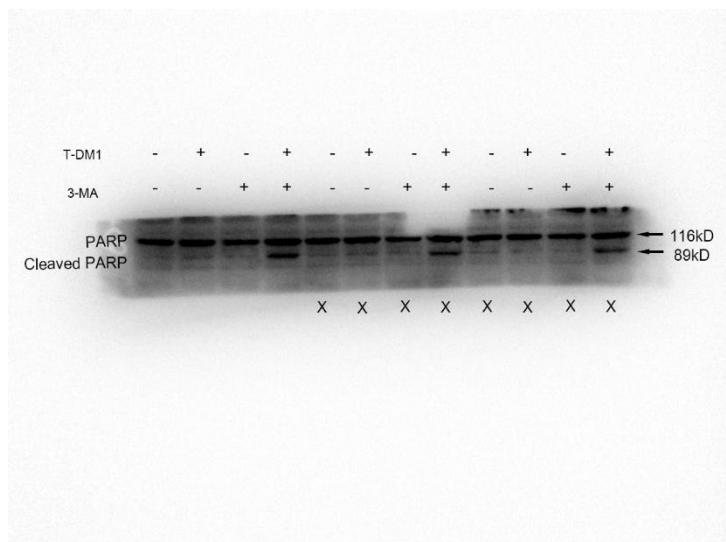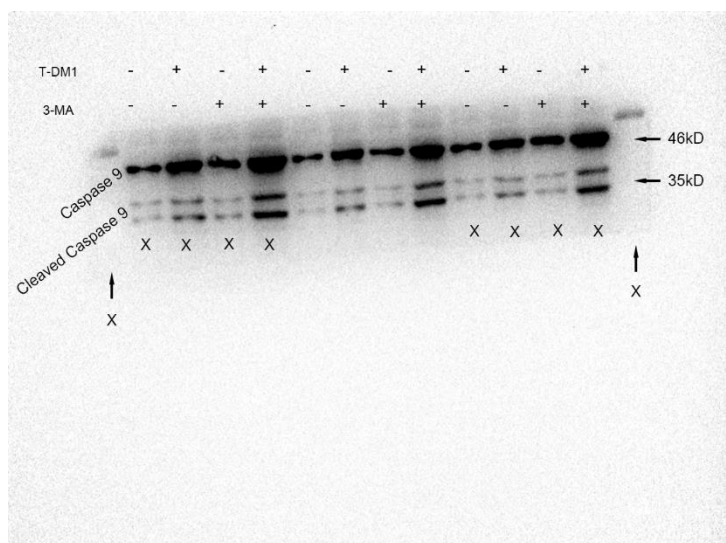

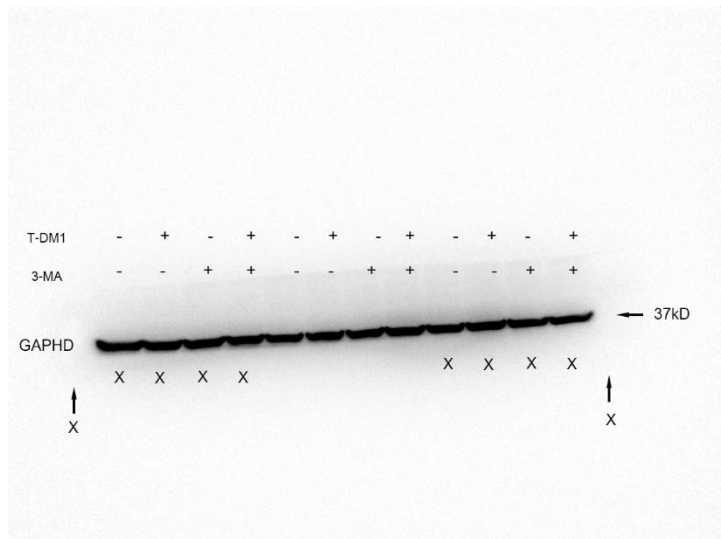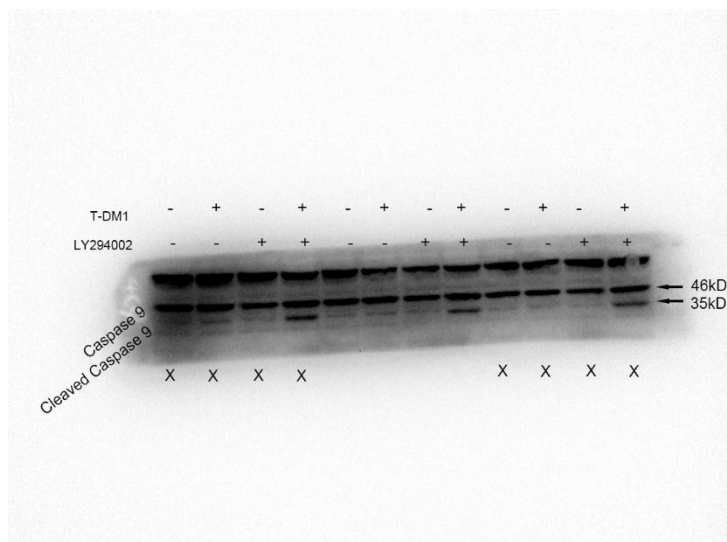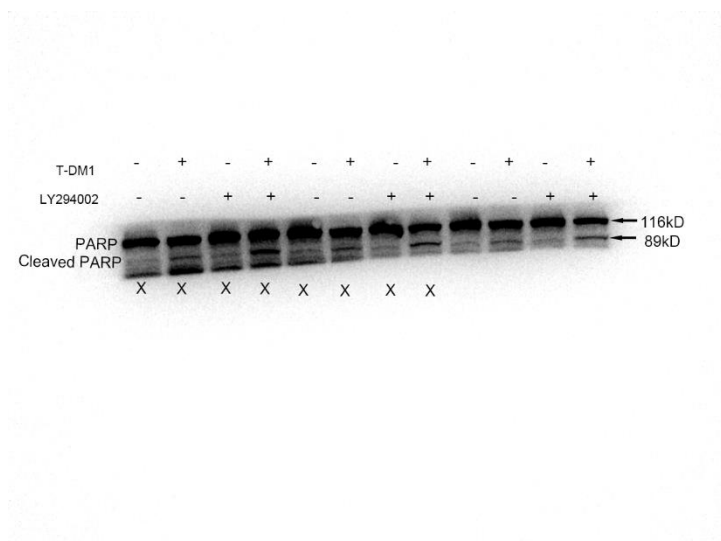

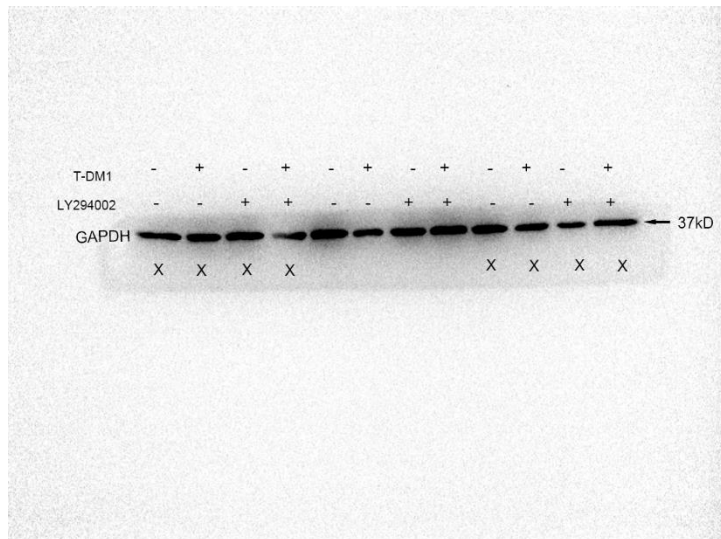

**Figure 5**

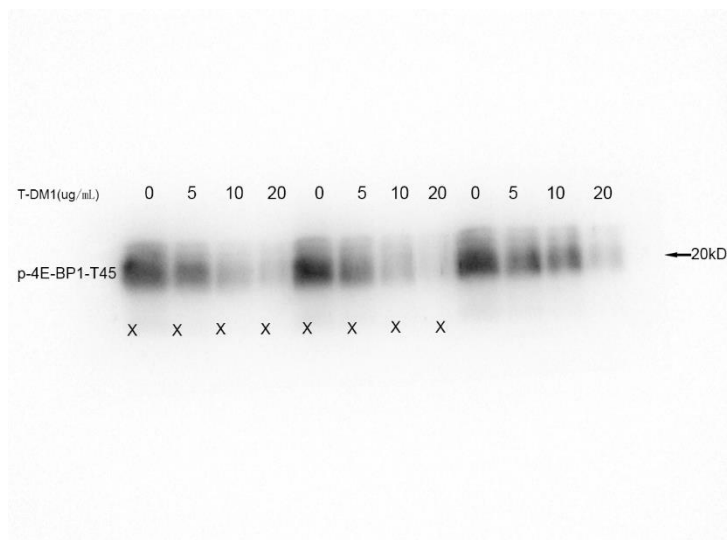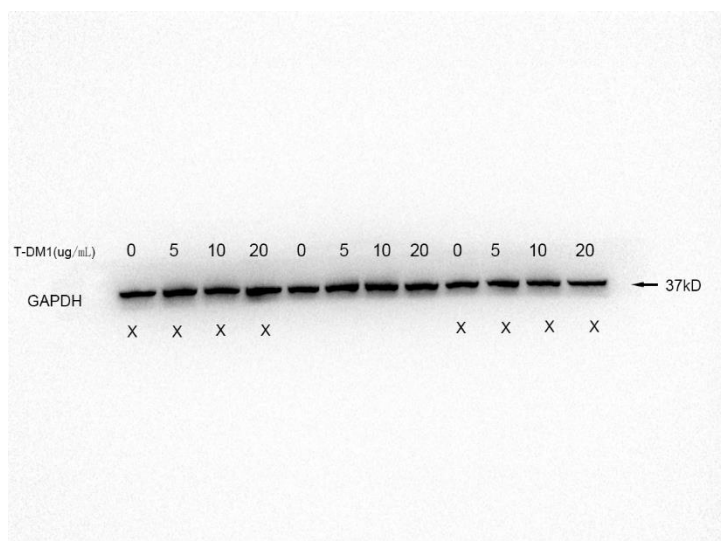

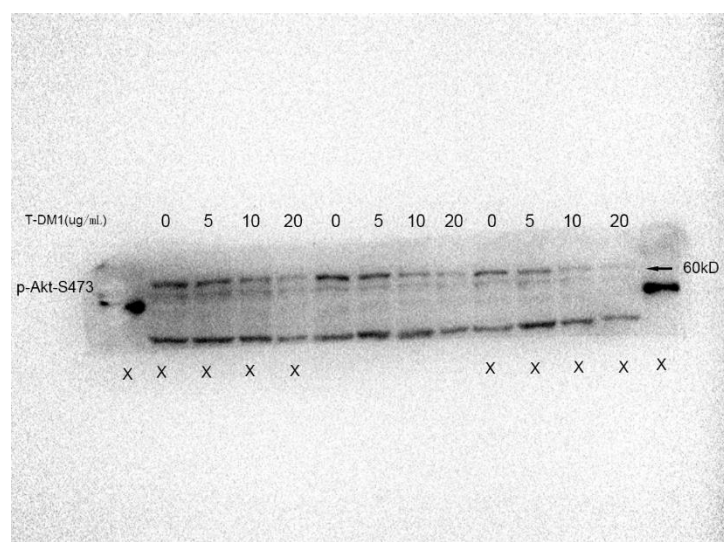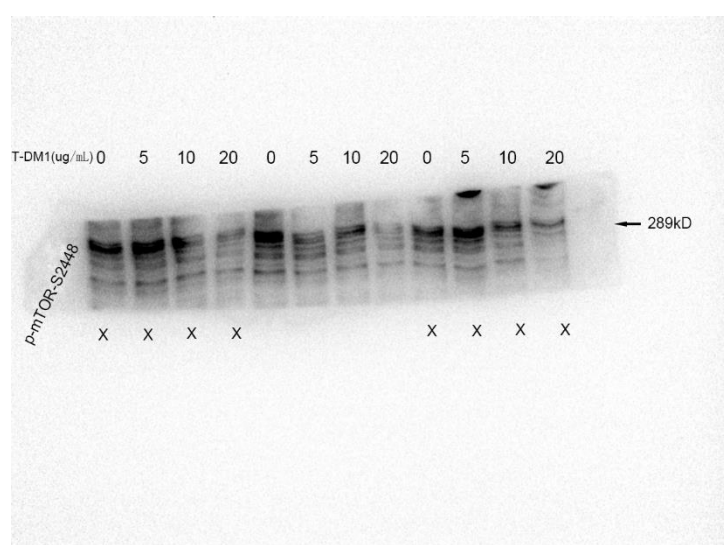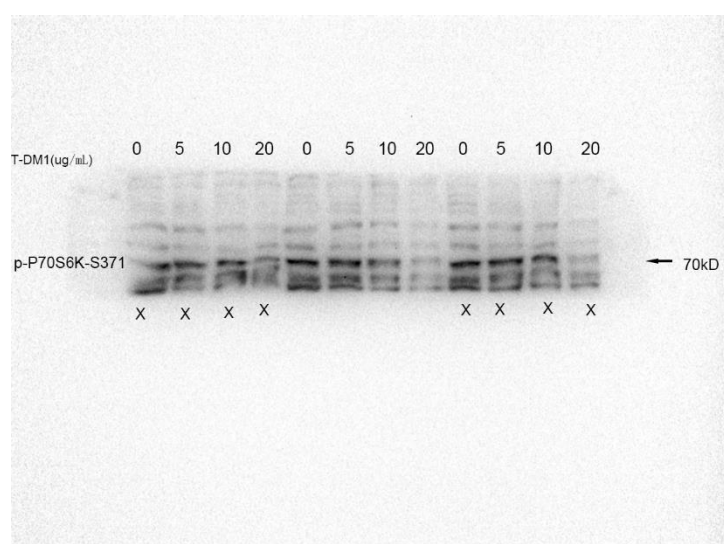

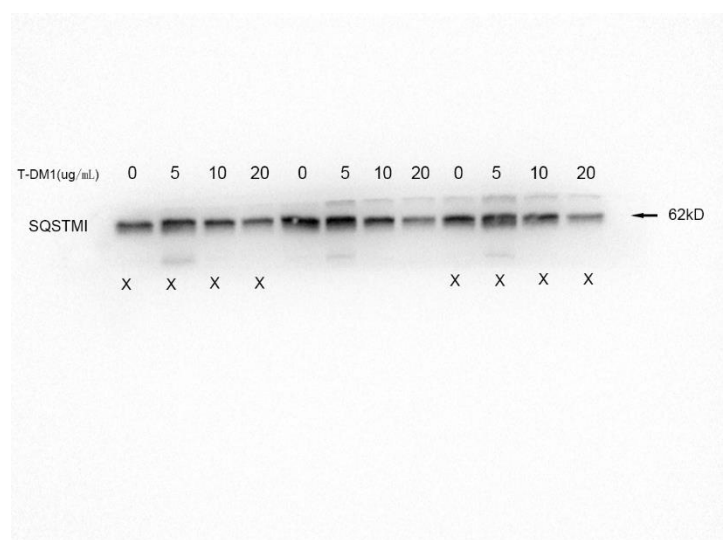

**Supplementary Figure S6**

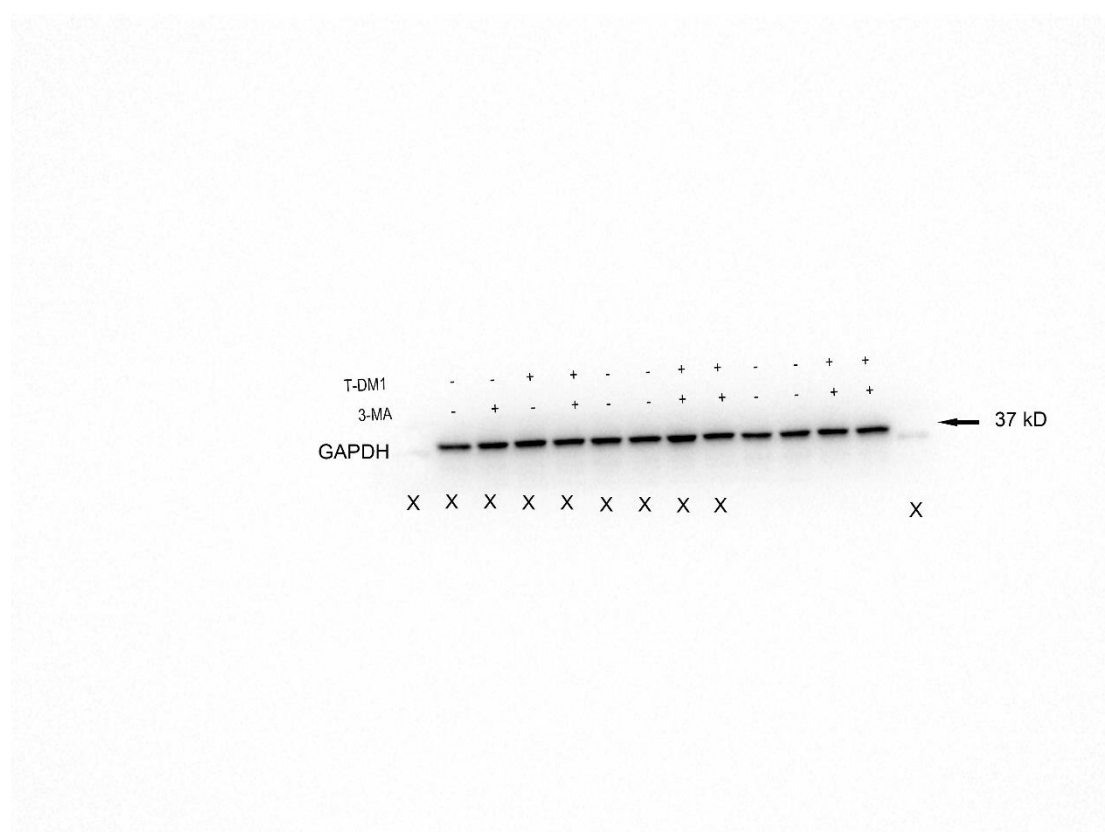

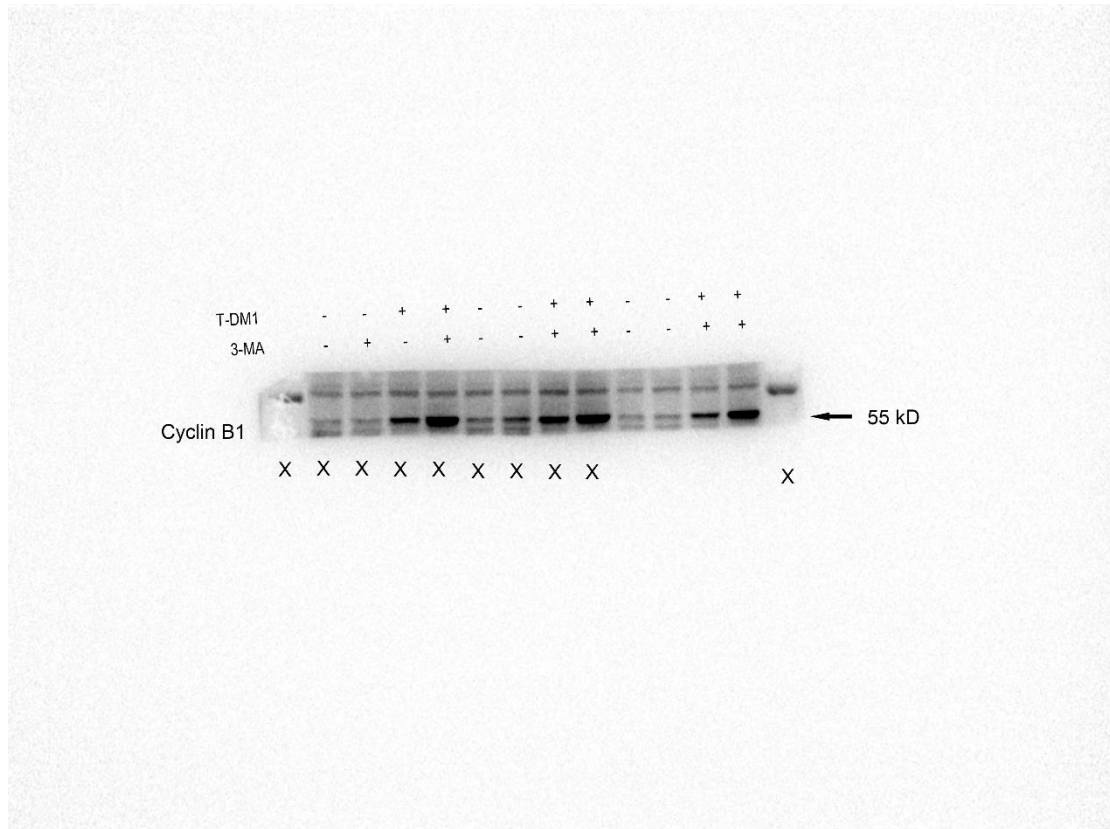

**Supplementary Figure S7**

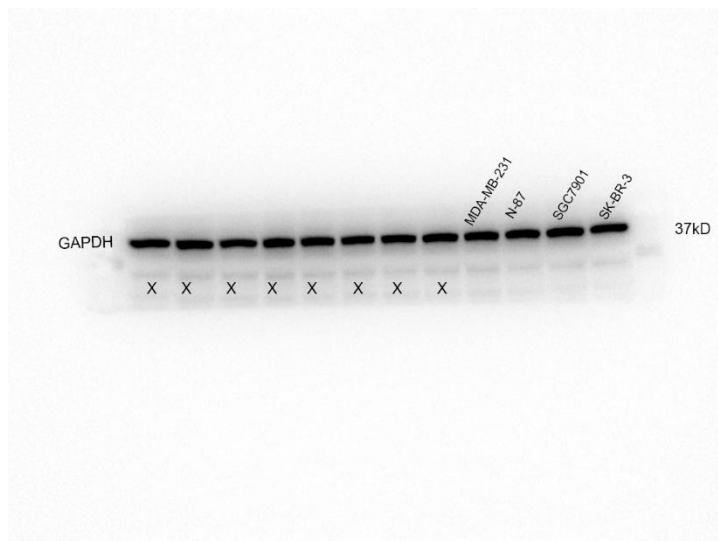

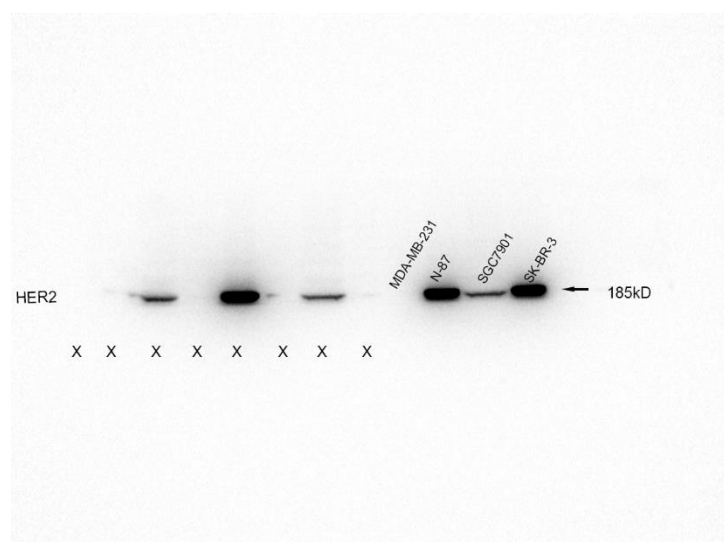

Supplement: S1 raw images — A supporting information caption for the file ‘S1_raw_images.pdf. (PDF) [file pone.0322029.s008.pdf]
