# Supplementary material for: Enhanced cytotoxicity of T-DM1 in HER2-low carcinomas via autophagy inhibition
Source: PLoS One. 2025 May 2;20(5):e0322029. doi: 10.1371/journal.pone.0322029 (PMC12047817; doi:10.1371/journal.pone.0322029)
Supplement: Attachment — Submitted filename: Comments.pdf [file pone.0322029.s009.pdf]

In this manuscript, Zhang and colleagues aimed to enhance the therapeutic efficacy of Ado-trastuzumab emtansine (T-DM1) in HER2-low gastric carcinoma cells, which typically exhibit limited responsiveness to this treatment. By investigating the role of autophagy in T-DM1 resistance, the authors discovered that T-DM1 induces cytoprotective autophagy via the Akt/mTOR pathway in HER2-low SGC7901 cells. This autophagy activation mitigates the drug's cytotoxic effects. However, pharmacological inhibition of autophagy significantly enhanced T-DM1-induced cytotoxicity and apoptosis by promoting lysosomal degradation of the drug and facilitating the release of its cytotoxic payload, emtansine. These findings highlight a novel strategy for improving T-DM1 efficacy in HER2-low gastric cancer by combining it with autophagy inhibitors, potentially broadening the therapeutic applications of T-DM1.

That said, there are several points that should be addressed before the manuscript is suitable for publication:

Major Comments:

1. In Figure 1A, the authors demonstrate that the HER2-low cell line exhibits limited responsiveness to T-DM1. To strengthen this claim, it would be beneficial to include a HER2-positive cell line as a control. A direct comparison of T-DM1 cytotoxicity between HER2-high and HER2-low cell lines would provide clearer evidence of the differential sensitivity.
2. The manuscript discusses the Akt/mTOR pathway's involvement in autophagy induced by T-DM1. However, to provide stronger mechanistic insight, the authors should consider using specific inhibitors of this pathway. This would validate whether the Akt/mTOR pathway is indeed a critical mediator of autophagy following T-DM1 treatment, as suggested in Figure 5.
3. While statistical analyses are mentioned, the manuscript lacks consistent information on the specific tests used for each figure. Including detailed descriptions of the statistical tests applied and the rationale for their selection would enhance the credibility and reproducibility of the findings.
4. The discussion could benefit from a more comprehensive comparison with existing studies on T-DM1 and other ADCs in HER2-low cancers, such as breast cancer. Additionally, elaborating on the broader therapeutic implications, such as the potential application of autophagy inhibitors in other HER2-low cancers or ADC therapies, would add significant value.

Minor Comments:

- The WB analysis for HER2 expression in Supplemental Figure 1 should be prioritized over immunofluorescence (IF) for its quantitative accuracy. Including a HER2-positive GC cancer cell line in the comparison panel would further enhance the relevance of the findings.
- There is some inconsistency in the use of terms such as "autophagy inhibition" and "blocking autophagy." Ensuring consistency in terminology throughout the manuscript would improve readability. A professional language review might also be beneficial to refine clarity and conciseness.

By addressing these points, the manuscript will be significantly strengthened and better positioned for publication. The study presents important insights into enhancing T-DM1 efficacy in HER2-low gastric cancer, and these revisions would further solidify the impact and rigor of the work.
