# Supplementary material for: Enhanced cytotoxicity of T-DM1 in HER2-low carcinomas via autophagy inhibition
Source: PLoS One. 2025 May 2;20(5):e0322029. doi: 10.1371/journal.pone.0322029 (PMC12047817; doi:10.1371/journal.pone.0322029)
Supplement: Attachment — Submitted filename: Response to Reviewers.docx [file pone.0322029.s011.docx]

Dear Prof. Ruo Wang,

We have studied the valuable comments from you, the assistant editor, and reviewers carefully, and tried our best to revise the manuscript. The point to point responds to the comments of reviewers are listed as following:

**Responses to the reviewers**

**Referee #1** (Remarks to the Author):

The manuscript explores the potential of combining T-DM1, an antibody-drug conjugate, with autophagy inhibitors to enhance its cytotoxicity in HER2-low gastric carcinoma cells. The authors concluded that T-DM1 alone exhibits limited cytotoxicity in HER2-low carcinoma cells due to the activation of cytoprotective autophagy. However, inhibiting autophagy pharmacologically significantly increases T-DM1’s cytotoxic effects by promoting its lysosomal fusion and facilitating the release of its active cytotoxic agent, emtansine.

Comments:

The study largely repurposes previously explored concepts by combining T-DM1 with autophagy inhibitors, an approach that has already been examined in other contexts. It does not provide groundbreaking insights or novel mechanisms specific to HER2-low gastric cancer.

Thank you very much for your constructive comments. Previous studies mainly focused on HER2-positive tumors, but our study focuses on HER2-low gastric cancer initially. In fact, the proportion of HER2-low gastric cancer (21%) is close to HER2-positive gastric cancer (23.2%), and groundbreaking insights or novel mechanisms specific need to be further studied (Nakayama I, Takahari D, Chin K, et al. Incidence, clinicopathological features, and clinical outcomes of low HER2 expressed, inoperable, advanced, or recurrent gastric/gastroesophageal junction adenocarcinoma. ESMO Open. 2023 Aug;8(4):101582.).

While the manuscript mentions the involvement of the Akt/mTOR pathway and autophagy modulation, it fails to thoroughly investigate the molecular interactions or provide sufficient evidence to establish causal relationships between autophagy inhibition and the enhanced efficacy of T-DM1.

Thanks very much for your valuable suggestion. The Akt/mTOR pathway is an important pathway regulating metabolism, proliferation and survival and plays a key role in maintaining cell homeostasis in cells. This signaling pathway can participate in the occurrence and development of many diseases by mediating autophagy. As a major regulatory molecule of cell growth and metabolism, mTOR can promote anabolic processes and inhibit catabolic processes (autophagy). Activated Akt can stimulate mTOR through a series of signal transduction processes, and ultimately inhibit autophagy. Our study suggests that the Akt/mTOR pathway and its downstream molecules were closely involved in the induction of autophagy by T-DM1 in SGC7901 cells. Furthermore, autophagy inhibition can significantly enhance T-DM1-mediated cytotoxicity and apoptosis. The modified contents were as follows.

“…Together, these results suggest that the Akt/mTOR pathway is restrained in the induction of autophagy by T-DM1 in SGC7901 cells.…”

The figures and results sections include excessive repetition, particularly in the presentation of apoptotic and autophagy-related assays. Streamlining this information would improve clarity and enhance the manuscript's readability.

Thanks very much for your valuable advice. Based on your suggestion, I simplified Figure 2. I placed the statistics from the FCM and Figure 2c into the supplementary data to improve the usability of the article.

The Figure 2. are modified and replaced appropriately.

Supplementary Figure S4. are added appropriately.

The modified contents were as follows.

“…**Figure 2** T-DM1 dose-dependently induced apoptosis in SGC7901 cells. **(A)** Apoptotic SGC7901 cells were stained with Annexin V-FITC/PI and observed after being incubated with T-DM1 for the indicated time (48 h) and were then examined by FCM. **(B)** Immunoblot analysis was used to measure the expression of apoptotic proteins (cleaved PARP, cleaved caspase 9, and cleaved caspase 3) after SGC7901 cells were treated with T-DM1 for 48 hours.…”

“…**Supplementary Figure S4.** **(A)** Apoptotic cells examined by FCM for the indicated time (48 h) were counted and analyzed, with results shown in bar charts (n = 3; mean ± S.D.; ****P* < 0.001). **(B-D)** Densitometric values of the expression of apoptotic proteins (cleaved PARP, cleaved caspase 9, and cleaved caspase 3) after SGC7901 cells treated with T-DM1 for 48 hours were calculated using ImageJ software and normalized to the value of the corresponding vehicle bands. The values of the vehicle group were adjusted to 1.0 and are shown as the mean ± S.D. Student’s t-test was used to analyze the data. **P* < 0.05 and ****P* < 0.001.…”

The manuscript lacks detailed descriptions of the statistical analyses performed, including criteria for significance and the sample sizes used for each experiment. This omission undermines the reproducibility and robustness of the findings.

Thank you for your advice. GraphPad Prism 8 (San Diego, CA) was used to analyze the data. The results were analyzed by Student’s t-test (n = 3 independent experiments) and are presented as the mean ± S.D. Differences were believed statistically significant when P < 0.05. According to your comment, we have added related data in our revised manuscript. The modified contents were as follows.

**Figure 1** T-DM1 exhibited limited cytotoxicity and was taken up by SGC7901 cells. **(A)** CCK-8 assays were used to evaluate the survival rates of SGC7901 cells treated with T-DM1 in a concentration-dependent manner for 72 hours (mean ± S.D.; ****P* < 0.001, n = 3).

The results were analyzed by Student’s t-test (n = 3 independent experiments). The sample size and mean ± S.D. in Figure 1A are shown in the table.

**Figure 3** Autophagosomes formation and autophagic flux were induced by T-DM1 in SGC7901 cells. **(A)** Immunoblot analysis showed the expression of autophagic proteins (SQSTM1 and LC3-Ⅱ) after SGC7901 cells were incubated with T-DM1 for 48 hours. The densitometric values were estimated by ImageJ software and normalized to the vehicle values. The data in the vehicle group were adjusted to 1.0, and data from three independent experiments are shown showed as the mean ± S.D. (Student’s t-test; **P* < 0.05; ****P* < 0.001, n = 3).

The results were analyzed by Student’s t-test (n = 3 independent experiments) and are presented as the mean ± S.D. Differences were believed statistically significant when P < 0.05.

**Figure 4** Autophagy inhibition induced by T-DM1 enhanced the cytotoxicity of SGC7901 cells. **(E)** Autophagy inhibition with 3-MA or LY294002 reduced the viability of SGC7901 cells, as determined by relevant kits (mean ± S.D.; **P* < 0.05 versus vehicle, n = 3). **(F)** The percentages of apoptotic SGC7901 cells were determined by FCM after incubation with 10 μg/ml T-DM1 and autophagy inhibitors (3-MA or LY294002). The results are presented in the side bar charts (****P* < 0.001, n = 3).

**Figure 5** The Akt/mTOR pathway and downstream components were involved in autophagy induced by T-DM1. **(B)** The densitometric values of SGC7901 cell samples were evaluated using ImageJ software and normalized to the corresponding values of the vehicle group. The values of the vehicle group were adjusted to 1.0 (mean ± S.D.; *P < 0.05; **P < 0.01 versus vehicle, n = 3).

The results were analyzed by Student’s t-test (n = 3 independent experiments) and are presented as the mean ± S.D. Differences were believed statistically significant when P < 0.05.

The results of Supplementary Material were analyzed by Student’s t-test (n = 3 independent experiments) and are presented as the mean ± S.D. Differences were believed statistically significant when P < 0.05.

The study does not adequately compare the efficacy of the proposed combination therapy with existing treatment options for HER2-low gastric cancer, limiting its clinical relevance and applicability.

Thank you for your advice. In our study, the effect of combination therapy was significantly better than that of single therapy for HER2-low gastric cancer in vitro experiments (Figure 4 E and F). Clinical relevance and applicability need to be further studied.

Phrases such as "significantly enhanced cytotoxicity" are used without providing clear quantitative benchmarks, leaving the practical significance of the findings unclear.

Thanks very much for your valuable advice. Based on your suggestion, I would be more careful to use "significantly enhanced cytotoxicity".

The discussion fails to place the findings within the broader landscape of HER2-low gastric cancer research. Conflicting evidence or alternative interpretations are not sufficiently addressed, leaving gaps in the contextual analysis.

Thank you for your suggestion. The modified contents were as follows.

“…GC, a prevalent malignant tumor, is receiving increasing attention because of its poor prognosis and high incidence [2]. HER2-targeted therapies are commonly used in clinical settings, but their application in HER2-low GC patients remains limited [4]. While HER2-targeted therapies have shown effectiveness in HER2+ GC, patients with HER2-low GC exhibit poor responses to these treatments [18, 19, 20]. Classification based on clinical features cannot fully describe biological characteristics, as nearly half of the HER2 negative BC tumors currently defined exhibit some degree of IHC expression, which has recently been renamed as HER2 low expression [21]. T-DM1, which is the first antibody-drug conjugate (ADC) approved for clinical use, is designed to decompose in lysosomes and disrupt microtubule organization, thereby selectively targeting HER2+ malignant tumor cells [20, 22, 23 , 24]. However, the effectiveness of T-DM1 is largely reduced in HER2-low tumors, so T-DM1 cannot be applied in clinical practice in HER2-low tumors. In recent years, people began to gradually pay attention to HER2-low gastric cancer, hoping to improve the effectiveness of targeted therapy [25]. This limited efficacy underscores the urgent need to better understand the mechanisms by which T-DM1 can be optimized for treating HER2-low GC.…”

“…ADCs represent a promising class of chemotherapy drugs that are engineered to deliver cytotoxic agents directly to target cells via monoclonal antibodies [36]. Ideally, target cells should remain highly sensitive to ADCs. However, our study showed that T-DM1 did not exhibit a superior therapeutic advantage against HER2-low GC cells [37]. Novel ADC drugs have been tested in clinical trials in HER2-low tumors, which suggests that fewer available HER2 receptors on cancer cells may be sufficient to achieve clinical benefits [38]. We observed the processing of T-DM1 from receptor recognition to intracellular lysosomes in SGC7901 cells, finding that fusion with lysosomes was strengthened by autophagy inhibition. Increased fusion of T-DM1 with lysosomes effectively increased the cytotoxic effect of T-DM1 on SGC7901 cells. Importantly, our findings, along with previous studies, suggest that autophagy interference in the dissociation of ADCs may be a universal phenomenon across different tumors [15, 39]. Therefore, regulating autophagy could potentially alter the sensitivity of ADC-based cancer treatment and could be a viable strategy to broaden the therapeutic range of ADCs.…”

The authors assert that autophagy inhibition may have universal applications across various tumor types but provide no supporting evidence beyond the context of HER2-low gastric cancer cells. This weakens the broader impact of the conclusions.

Thanks very much for your valuable suggestion. In a previous study, we found that autophagy occurs extensively during the treatment of tumors. The study of Zhang J, et al. showed that targeting the autophagy promoted antitumor effect of T-DM1 on HER2-positive Gastric Cancer (Zhang J, et al. Targeting the autophagy promoted antitumor effect of T-DM1 on HER2-positive gastric cancer. Cell Death Dis. 2021 Mar 17;12(4):288. doi: 10.1038/s41419-020-03349-1.). Another paper by Shen, W. et al. also revealed the same antitumor effect in non-small cell lung cancer (Shen, W. et al. A novel and promising therapeutic approach for NSCLC: recombinant human arginase alone or combined with autophagy inhibitor. Cell Death Dis. 2017 Mar 30;8(3): e2720. doi: 10.1038/cddis.2017.137.). We will add the corresponding references to reinforce the conclusion of the article. The modified contents were as follows.

“…Importantly, our findings, along with previous studies, suggest that autophagy interference in the dissociation of ADCs may be a universal phenomenon across different tumors [15, 36]. Therefore, regulating autophagy could potentially alter the sensitivity of ADC-based cancer treatment. To date, it remains unclear whether modulating autophagy could be a viable strategy to broaden the therapeutic range of ADCs. …”

**Referee #2** (Remarks to the Author):

In the manuscript entitled: “Enhanced cytotoxicity of T-MD1 in HER2-low carcinomas via autophagy inhibition”, Zhang et al. showed that T-DM1 induces autophagy and combination of T-DM1 with autophagy inhibitors increases T-DM1 efficacy in HER2-low gastric cancer. A potential expansion of the use of T-DM1 and may be other HER2-targeted therapies to tumors with low HER2 expression is significant since the HER2-low patient subpopulation usually exhibits an aggressive disease progression. Despite that, there are several major points that need to be addressed as listed below:

Major points:

1- All the IF images need quantification.

Thanks very much for your valuable suggestion. All the IF images had been quantified. This can be seen in Supplementary Figure S1, Supplementary Figure S2. (B-D) and Supplementary Figure S5. The modified contents were as follows.

“…The brightness values of HER2 red dye staining were estimated using ImageJ software and the densitometric values were normalized to the corresponding values of the vehicle group. The values of the vehicle group were set as 1.0 (n = 3 independent experiments; mean ± S.D.; ***P* < 0.01 and ****P* < 0.001). …”

“…(C) The brightness values of the Alexa Fluor™ 647 were evaluated using ImageJ software, and the values were normalized to the corresponding values of the vehicle group. **(D)** …”

“…**Supplementary Figure S5.** The brightness values of the Alexa Fluor™ 647 visualized by red fluorescence, Cyto-ID visualized by fluorescence and LysoTracker exhibited yellow fluorescence were evaluated using ImageJ software, and the values were normalized to the corresponding values of the vehicle group. …”

2- In Fig. 1B, it was claimed that endocytosis happens at 6 hrs, however, it is not visible from the image provided.

Thank you very much for your constructive comments. we claimed that endocytosis happening at 6 hrs was based on the uniform distribution of the labeling T-DM1 with red fluorescence on the cells. This conclusion really lacks more accurate experimental support, so we have made corresponding modifications to the paper. The modified contents were as follows.

“…Laser confocal microscopy was used to track the uptake of T-DM1 at different time points, revealing that T-DM1 bound to the HER2 receptor at 1 hour and internalized at 12 hours post-treatment (Figure 1B). …”

3- Is there any change in T-DM1 internalization upon combination of T-DM1 with autophagy inhibitors.

Thank you for your asking. We speculated that T-DM1 internalization upon combination of T-DM1 with autophagy inhibitors should be enhanced, thus enhancing cytotoxicity (Figure 6 and Supplementary Figure S5).

4- It is not clear how the apoptotic cells were quantified. Were they the Annexin V+ and PI- cells corresponding to early apoptosis or Annexin V+ PI+ cells corresponding to late apoptosis?

Thank you very much for your constructive comments. We quantitatively counted early apoptosis (the Annexin V+ and PI- cells). The modified contents were as follows.

“…**(F)** The percentages of early apoptotic SGC7901 cells (the Annexin V+ and PI- cells) were determined by FCM after incubation with 10 μg/ml T-DM1 and autophagy inhibitors (3-MA or LY294002). The results are presented in the side bar charts (****P* < 0.001, n = 3).…”

“…Apoptotic cells (the Annexin V+ and PI- cells) examined by FCM for the indicated time (48 h) were counted and analyzed, with results shown in bar charts (n = 3; mean ± S.D.; ****P* < 0.001).…”

5- Is DM1 treatment also inducing autophagy induction upon mTOR inhibition?

Thank you for your asking. Our current experimental results can only prove than the Akt/mTOR pathway is restrained in the induction of autophagy by T-DM1 in SGC7901 cells. The study of Zhang J, et al. showed that T-DM1 induced cytoprotective autophagy by inactivating the Akt/mTOR signaling pathway (Zhang J, et al. Targeting the autophagy promoted antitumor effect of T-DM1 on HER2-positive gastric cancer. Cell Death Dis. 2021 Mar 17;12(4):288. doi: 10.1038/s41419-020-03349-1.).

6- The reduction of cell viability upon combination of T-DM1 with the autophagy inhibitors is not convincing.

Thank you very much for your comments. LY294002 or 3-MA enhanced T-DM1-induced cytotoxicity in SGC7901 cells (Figure 4E). FCM results confirm the above experimental results, which demonstrated a notable increase in the number of apoptotic cells when combining T-DM1 with LY294002 or 3-MA (Figure 4F). We verified our experiment again and got similar experimental results, as shown in the picture.

7- Since the major mechanism of action of T-DM1 is mitotic arrest, it is suggested to examine the mitotic arrest markers in HER2-low cells treated with T-DM1 and autophagy inhibitor combination.

Thank you very much for your constructive comments. SGC7901 cells were incubated with T-DM1 and 3-MA for 48 hours, and cell lysates were analyzed by immunoblotting to determine the expression levels of the CycinB1. It was found that, alongside treated with T-DM1 and autophagy inhibitor combination, CycinB1 which was the mitotic arrest markers was increased. The modified contents were as follows.

“…Supplementary Figure S6. SGC7901 cells were incubated with T-DM1 and 3-MA for 48 hours, and cell lysates were analyzed by immunoblotting to determine the expression levels of the CycinB1. The densitometric values of SGC7901 cell samples were evaluated using ImageJ software and normalized to the corresponding values of the vehicle group. The values of the vehicle group were adjusted to 1.0 (mean ± S.D.; *P < 0.05; **P < 0.01 versus vehicle, n = 3).…”

8- To eliminate the potential cell line-specific effects, it is suggested that the key experiments, e.g., reduced cell viability and apoptosis induction upon combination of T-DM1 with the autophagy inhibitors are repeated in a second HER2-low gastric cancer cell line.

Thanks very much for your valuable suggestion. It is difficult for our laboratory to obtain another HER2-low gastric cancer cell line. But the key experiment, e.g., reduced cell viability and apoptosis induction upon combination of T-DM1 with the autophagy inhibitors are repeated in a second HER2-positive gastric cancer cell line (N87), which corresponding experimental data had been published in Cell Death Dis. (Zhang J, et al. Targeting the autophagy promoted antitumor effect of T-DM1 on HER2-positive gastric cancer. Cell Death Dis. 2021 Mar 17;12(4):288. doi: 10.1038/s41419-020-03349-1.).

Reviewer #3: In this manuscript, Zhang and colleagues aimed to enhance the therapeutic efficacy of Ado-trastuzumab emtansine (T-DM1) in HER2-low gastric carcinoma cells, which typically exhibit limited responsiveness to this treatment. By investigating the role of autophagy in T-DM1 resistance, the authors discovered that T-DM1 induces cytoprotective autophagy via the Akt/mTOR pathway in HER2-low SGC7901 cells. This autophagy activation mitigates the drug's cytotoxic effects. However, pharmacological inhibition of autophagy significantly enhanced T-DM1-induced cytotoxicity and apoptosis by promoting lysosomal degradation of the drug and facilitating the release of its cytotoxic payload, emtansine. These findings highlight a novel strategy for improving T-DM1 efficacy in HER2-low gastric cancer by combining it with autophagy inhibitors, potentially broadening the therapeutic applications of T-DM1.

That said, there are several points that should be addressed before the manuscript is suitable for publication:

Major Comments:

1. In Figure 1A, the authors demonstrate that the HER2-low cell line exhibits limited responsiveness to T-DM1. To strengthen this claim, it would be beneficial to include a HER2-positive cell line as a control. A direct comparison of T-DM1 cytotoxicity between HER2-high and HER2-low cell lines would provide clearer evidence of the differential sensitivity.

Thanks very much for your valuable suggestion. We have completed the corresponding experiments and obtained reliable experimental results. The modified contents were as follows.

“…Supplementary Figure S1. (C**)** CCK-8 assays were used to evaluate the survival rates of SK-BR-3 cells (HER2-high Breast cancer cells) treated with T-DM1 in a concentration-dependent manner for 72 hours (mean ± S.D.; ***P < 0.001, n = 3).…”

2. The manuscript discusses the Akt/mTOR pathway's involvement in autophagy induced by T-DM1. However, to provide stronger mechanistic insight, the authors should consider using specific inhibitors of this pathway. This would validate whether the Akt/mTOR pathway is indeed a critical mediator of autophagy following T-DM1 treatment, as suggested in Figure 5.

Thanks very much for your valuable suggestion. We performed genetic tests on SGC7901 cells intervened or without intervened by T-DM1, which found that the Akt/mTOR pathway is a critical mediator of autophagy following T-DM1 treatment.

3. While statistical analyses are mentioned, the manuscript lacks consistent information on the specific tests used for each figure. Including detailed descriptions of the statistical tests applied and the rationale for their selection would enhance the credibility and reproducibility of the findings.

Thanks very much for your valuable suggestion. GraphPad Prism 8 (San Diego, CA) was used to analyze the data. The results were analyzed by Student’s t-test (n = 3 independent experiments) and are presented as the mean ± S.D. Differences were believed statistically significant when P < 0.05. The statistical methods, criteria for significance and the sample sizes of each experiment were significantly labeled in the article.

4. The discussion could benefit from a more comprehensive comparison with existing studies on T-DM1 and other ADCs in HER2-low cancers, such as breast cancer. Additionally, elaborating on the broader therapeutic implications, such as the potential application of autophagy inhibitors in other HER2-low cancers or ADC therapies, would add significant value.

Thank you for your suggestion. The modified contents were as follows.

“…GC, a prevalent malignant tumor, is receiving increasing attention because of its poor prognosis and high incidence [2]. HER2-targeted therapies are commonly used in clinical settings, but their application in HER2-low GC patients remains limited [4]. While HER2-targeted therapies have shown effectiveness in HER2+ GC, patients with HER2-low GC exhibit poor responses to these treatments [18, 19, 20]. Classification based on clinical features cannot fully describe biological characteristics, as nearly half of the HER2 negative BC tumors currently defined exhibit some degree of IHC expression, which has recently been renamed as HER2 low expression [21]. T-DM1, which is the first antibody-drug conjugate (ADC) approved for clinical use, is designed to decompose in lysosomes and disrupt microtubule organization, thereby selectively targeting HER2+ malignant tumor cells [20, 22, 23 , 24]. However, the effectiveness of T-DM1 is largely reduced in HER2-low tumors, so T-DM1 cannot be applied in clinical practice in HER2-low tumors. In recent years, people began to gradually pay attention to HER2-low gastric cancer, hoping to improve the effectiveness of targeted therapy [25]. This limited efficacy underscores the urgent need to better understand the mechanisms by which T-DM1 can be optimized for treating HER2-low GC.…”

“…ADCs represent a promising class of chemotherapy drugs that are engineered to deliver cytotoxic agents directly to target cells via monoclonal antibodies [36]. Ideally, target cells should remain highly sensitive to ADCs. However, our study showed that T-DM1 did not exhibit a superior therapeutic advantage against HER2-low GC cells [37]. Novel ADC drugs have been tested in clinical trials in HER2-low tumors, which suggests that fewer available HER2 receptors on cancer cells may be sufficient to achieve clinical benefits [38]. We observed the processing of T-DM1 from receptor recognition to intracellular lysosomes in SGC7901 cells, finding that fusion with lysosomes was strengthened by autophagy inhibition. Increased fusion of T-DM1 with lysosomes effectively increased the cytotoxic effect of T-DM1 on SGC7901 cells. Importantly, our findings, along with previous studies, suggest that autophagy interference in the dissociation of ADCs may be a universal phenomenon across different tumors [15, 39]. Therefore, regulating autophagy could potentially alter the sensitivity of ADC-based cancer treatment and could be a viable strategy to broaden the therapeutic range of ADCs.…”

Minor Comments:

- The WB analysis for HER2 expression in Supplemental Figure 1 should be prioritized over immunofluorescence (IF) for its quantitative accuracy. Including a HER2-positive GC cancer cell line in the comparison panel would further enhance the relevance of the findings.

Thanks very much for your valuable suggestion. We have completed the corresponding experiments and obtained reliable experimental results. The modified contents were as follows.

“…**Supplementary Figure S7.** The cell lysates of MDA-MB-231 (HER2-negative Breast cancer cell line), SGC7901 and N-87 cells (HER2-positive GC cancer cell line) were analyzed by immunoblotting to determine the expression levels of the HER2.…”

- There is some inconsistency in the use of terms such as "autophagy inhibition" and "blocking autophagy." Ensuring consistency in terminology throughout the manuscript would improve readability. A professional language review might also be beneficial to refine clarity and conciseness.

Thanks very much for your valuable suggestion and guidance for my writing. I have revised the whole text and standardized the wording.

By addressing these points, the manuscript will be significantly strengthened and better positioned for publication. The study presents important insights into enhancing T-DM1 efficacy in HER2-low gastric cancer, and these revisions would further solidify the impact and rigor of the work.

I really appreciate your consideration of our manuscript, and look forward to receiving feedback. If you have any queries, please don’t hesitate to contact me.

Yours sincerely,

Qun Xin, Ph.D., Professor

Department of General Surgery

Naval 971 Hospital of Chinese People's Liberation Army
